# Antimicrobial Activity and GC-MS Profile of Copaiba Oil for Incorporation into *Xanthosoma mafaffa* Schott Starch-Based Films

**DOI:** 10.3390/polym12122883

**Published:** 2020-12-01

**Authors:** Giovana de Menezes Rodrigues, Cristina Tostes Filgueiras, Vitor Augusto dos Santos Garcia, Rosemary Aparecida de Carvalho, José Ignacio Velasco, Farayde Matta Fakhouri

**Affiliations:** 1 Faculty of Engineering, Federal University of Grande Dourados, Dourados 79804-970, MS, Brazil; giovanademr@gmail.com (G.d.M.R.); cristinafilgueiras@ufgd.edu.br (C.T.F.); garcia.vitoraugusto@gmail.com (V.A.d.S.G.); 2 Faculty of Animal Science and Food Engineering, University of São Paulo, Av. Duque de Caxias Norte, 225, Pirassununga 13635-900, SP, Brazil; rosecarvalho@usp.br; 3 Poly 2 Group, Department of Materials Science and Engineering, Universitat Politècnica de Catalunya (UPC BarcelonaTech), ESEIAAT, Carrer de Colom, 11, 08222 Terrassa, Spain; jose.ignacio.velasco@upc.edu

**Keywords:** *Copaifera* sp., complex coacervation, antimicrobial activity, unconventional starch, biodegradable packaging

## Abstract

The present study evaluated the effect of the incorporation of copaiba oil, in direct and in microencapsulated form, into films based on *Xanthosoma mafaffa* Schott starch. Initially, the characterization of copaiba oil by gas chromatograph coupled with mass spectrometry (GC-MS) and its antimicrobial activity against gram-positive and gram-negative bacteria was performed. The films were produced by the casting technique and characterized in relation to physical, chemical, structural, and antimicrobial activity. Sesquiterpenes, mainly β-caryophyllene, were the predominant compounds in copaiba oil, showing antimicrobial activity against *B. subtilis* and *S. aureus*. The films showed forming capacity, however, was observed a decrease in solubility and revealed an increase in hydrophobic characteristics. However, the oil reduced the tensile strength and elongation, while the microcapsules did not influence the mechanical properties in comparison to the control film. From microstructure analysis, changes in the films roughness and surface were observed after the addition of oil both directly and in microencapsulated form. Films incorporated with microparticles were able to inhibit the gram-positive bacteria tested, forming inhibition zones, indicating that the encapsulation of copaiba oil was more efficient for protecting bioactive compounds from the oil, suggesting the possible application of mangarito starch-based films incorporated with copaiba oil as biodegradable packaging.

## 1. Introduction

Biodegradable films or edible coatings appear as an alternative to reduce environmental impacts caused by petroleum-derived polymers [1] and the production of starch-based films is advantageous as it is considered a renewable polymer, has a biodegradable nature, and is available in several types of plants [2]. Considered the main reserve carbohydrate in plants, starch is composed mainly of amylose and amylopectin and plays an important role in the food industry by providing stability, viscosity, and consistency when applied to food products [3,4].

Due to the extensive application of starch in product development, there is a growing interest from the industry in finding new sources of this polymer with a high content of amylose, amylopectin, and good thermal properties [4,5]. Thus, studies in the literature report obtaining starch from non-conventional sources, such as sorghum grains, mung beans and peas [5], yams [6], arrowroot [7], arracacha [8], taro [9], and mangarito [10].

Mangarito (*Xanthosoma mafaffa* Schott) is a non-conventional vegetable of cultural importance, appreciated for its leaves and edible rhizomes [11]. Rhizomes are considered a source of carbohydrates, which enables the extraction of starch for the production of biodegradable films or edible coatings [10,12]. According to Ávila et al. [10], biodegradable films based on mangarito starch are homogeneous, transparent, and flexible. 

Starch-based films generally have a hydrophilic character, which causes undesirable changes to their mechanical and barrier properties [13]. According to Atarés and Chiralt [14], the addition of essential oils can contribute to reducing the hydrophilicity of films and promote the development of active biodegradable packaging with antimicrobial properties. According to Leandro et al. [15], the bioactivity of copaiba oil can be confirmed mainly by the presence of sesquiterpenes, with emphasis on β-caryophyllene. In addition, this oil is considered a natural food additive that can be consumed pure or with food, according to the Food and Drug Administration [16]. 

Brandelero et al. [17] studied the incorporation of copaiba essential oil into films made of cassava starch, polyvinyl alcohol and sodium alginate, plasticized with glycerol, and reported that the films showed antifungal activity against *Fusarium* sp. The same effect was reported by Morelli et al. [18], who coated bio-based materials (paper and poly lactic acid) with copaiba oil and detected antimicrobial activity against *Bacillus subtilis*. 

The incorporation of essential oil into films is usually carried out by the emulsion between the oil and other components of the filmogenic solution, with the aid of a homogenizer [19] and the use, or not, of stabilizing agents [20], or encapsulated oil [21]. However, the incorporation of essential oils can affect the structural, barrier, mechanical, and optical properties of films [14,22].

One of the alternatives is microencapsulation, which can assist in maintaining the bioactive components of essential oils [23]. This technique provides protection, stability, and allows the gradual release of these compounds [24]. However, scientific reports about the production of microparticles of copaiba oil incorporated into mangarito starch-based films are incipient in the literature. In this context, the present work aimed to evaluate methods and the effects of incorporating copaiba oil into mangarito starch-based films on the physical, chemical, structural, and antimicrobial properties. 

## 2. Materials and Methods 

### 2.1. Material

The material used to produce the films was mangarito starch (*Xanthosoma mafaffa* Schott, Dourados, Brazil) composed of 7.91 ± 0.11% moisture, 0.15 ± 0.06% ash, 0.03 ± 0.03% lipids, 2.24 ± 0.02% protein, 89.58 ± 0.13% carbohydrates, and 25.78 ± 0.49% amylose, obtained from rhizomes harvested in the experimental crop 2017–2018 of the Medicinal Plants Garden of the Federal University of Grande Dourados, Dourados, Brazil, commercial copaiba oil (Verde Nattus, Maringá, Brazil) purchased at Mundo Verde in Dourados/MS, and glycerol (Dynamic, Indaiatuba, Brazil). Gelatin type A, Bloom 240, GAP 6 (Gelita^®^ from Brazil, Cotia, Brazil), gum arabic (Dynamic, 85%, Indaiatuba, Brazil), and hydrochloric acid (LS Chemicals, 37%, Ribeirão Preto, Brazil) were used in the production of microparticles. For the antimicrobial activity, the following bacterial strains were used: *Bacillus subtilis* (ATCC WDCM 00003), *Staphylococcus aureus* (ATCC 25923), *Escherichia coli* (ATCC WDCM00013), *Pseudomonas aeruginosa* (ATCC 27853), *Enterobacter aerogenes* (ATCC 13048) and *Salmonella typhimurium* (ATCC WDCM 00031) donated by Microbiology Laboratory from National Service for Industrial Training (Dourados, Brazil), Brain Heart Infusion (BHI) broth (Prodimol Biotechnology, Belo Horizonte, Brazil), Mueller–Hinton agar (Kasvi, São José dos Pinhais, Brazil), dimethyl sulfoxide solvent (Vetec, 99.9%, Duque de Caxias, Brazil), chloramphenicol antibiotic (Lb Laborclin, 30 μg, Pinhais, Brazil), and a paper filter disc (J. Probal, Qualy, pores 14 μm, São José dos Pinhais, Brazil).

### 2.2. Methods

#### 2.2.1. Chemical Composition of Copaiba Oil

The chemical composition of the copaiba oil was determined in a gas chromatograph coupled with mass spectrometry (CG-EM, GCMS-QP2010 Ultra, Shimadzu, Kyoto, Japan), using a capillary column DB-5 (30 m × 0.25 mm × 0.25 μm), programmed at an initial temperature of 50 °C, increasing at a rate of 3 °C min^−1^, up to 280 °C. The flow rate of the carrier gas was 1.0 mL min^−1^. Analyzes were performed with the injector temperature at 220 °C and the detector temperature at 290 °C, using an injection volume of 1 µL, in 1:10 split mode. For the sample preparation, the oil was diluted in n-hexane (100 µg mL^−1^). The calculated retention index (CI) was determined using the linear alkanes standard (C7-C40, Sigma Aldrich, 98%), and the identification of the compounds was obtained by comparing the CI with the literature retention index (LI) [25] and interpretation of the mass spectra of the samples and a comparison with the databases (NIST21 and WILLEY 229). 

#### 2.2.2. Zone of Inhibition Test

The zone of inhibition test was carried out according to the standardized methodology National Committee for Clinical Laboratory Standards [26] in order to verify the antimicrobial activity of copaiba oil against *Staphylococcus aureus*, *Bacillus subtilis*, *Escherichia coli*, *Pseudomonas aeruginosa*, *Enterobacter aerogenes*, and *Salmonella typhimurium*. Initially, the culture was activated in 5 mL of BHI broth and incubated in a bacteriological oven (Solab, SL-101, Piracicaba, Brazil) at 35 ± 2 °C for 18 h. Then, the absorbance of the inoculum was determined in a spectrophotometer (Marconi, Jenway 7310, Staffordshire, UK) at 675 nm, in which the suspension turbidity was adjusted to a 0.5 McFarland standard solution (10^8^ CFU mL^−1^). Then, 100 µL of the inoculum was seeded on plates containing Mueller–Hinton agar culture medium and filter paper discs (5.5 mm in diameter), impregnated with copaiba oil, were added. The plates were incubated in a bacteriological oven (Solab, SL-101, Piracicaba, Brazil) at 35 ± 2 °C for 24 h. After this period, microbial growth was observed and the degree of inhibition was expressed as inhibition zone (mm).

#### 2.2.3. Minimum Inhibitory Concentration (MIC)

The minimum inhibitory concentration (MIC) was determined using the disk diffusion technique [27]. The tests were performed using only the strains inhibited in the zone of inhibition test. The cultures were activated and the plates were prepared as described in Section 2.2.2. The filter paper discs (5.5 mm in diameter) were impregnated with oil diluted in dimethyl sulfoxide in sterile tubes containing a total volume of 1 mL, in the following concentrations: 3, 6, 12, 25, 50, and 100% of copaiba oil and a period of 1 min was required for the paper disc to be impregnated with the respective concentrations of copaiba oil. Then, the impregnated discs were placed on plates containing the seeded inoculum and were incubated in a bacteriological incubator at 35 ± 5 ° C for 24 h. After this period, microbial growth and MIC with an inhibition zone of ≥ 8 mm were detected [28,29]. The antibiotic chloramphenicol was used as the positive control while the dimethyl sulfoxide solvent was used as the negative control.

### 2.3. Production of Microparticles with Copaiba Oil

The microparticles were produced by complex coacervation based on the methodology proposed by Alvim and Grosso [30]. Initially, 5 g of copaiba oil was homogenized in Ultra-Turrax (IKA, T25 Digital Ultra-Turrax, Staufen, Germany), at 14.000 rpm for 3 min, in 100 mL of gelatin solution (2.5 g of gelatin/100 mL of distilled water) at 45 °C (IKA, C-MAG HS7, Staufen, Germany). Then, 100 mL of gum arabic solution (2.5 g of gum arabic/100 mL of distilled water) was added to the emulsion under magnetic stirring at 250 rpm for 10 min with the same temperature. Subsequently, 400 mL of distilled water (45 °C) was added and the mixture remained under magnetic stirring for 5 min. The pH was then adjusted to 4.0 using hydrochloric acid solution (2.7 M) and the mixture was cooled under magnetic stirring for 1 h, using an ice bath (until reaching 10 ± 2 °C). Subsequently, the mixture was kept at 4 °C overnight (5 ± 2 °C) for a complete precipitation of the microparticles. These were separated from the solution using a Tyler 200 mesh sieve and stored in glass flasks at −22 °C.

### 2.4. Production of Films

The films were produced by the casting technique, using mangarito starch (3 g/100 g of filmogenic solution) and glycerol (0.9 g/100 g of filmogenic solution). The copaiba oil concentration for direct incorporation was defined according to the MIC, and the concentration of microparticles (10 g/100 g of filmogenic solution) was defined through preliminary tests.

Initially, mangarito starch and glycerol were dispersed in distilled water under mechanical stirring (IKA, Color Squid, Staufen, Germany) at 200 rpm, using a thermostatic bath (Marconi, MA 127, Piracicaba, Brazil) at 97 °C for 20 min. For the incorporation of copaiba oil and microparticles, the filmogenic solution was cooled down to 45 °C. The copaiba oil was added using Ultra-Turrax (IKA, T25 Digital Ultra-Turrax, Staufen, Germany), for 4 min at 10.000 rpm [19], while the microparticles were incorporated under magnetic stirring (IKA, Color Squid, Staufen, Germany) at 500 rpm (20 min). In addition, films incorporated with copaiba oil, directly or microencapsulated, were produced without the addition of plasticizer, with the control film being composed of mangarito starch and glycerol. The filmogenic solutions were dispersed on acrylic plates (12 cm × 12 cm) and dried for 24 h at 30 °C in an air circulating oven (Marconi, MA035, Piracicaba, Brazil). After this period, the films were packed in desiccators (Nalgon, 810, Itupeva, Brazil) containing NaBr (58% RH, Dynamic, Indaiatuba, Brazil). 

### 2.5. Caracterization of Films 

#### 2.5.1. Visual Analysis, Scanning Electron Microscopy, and Atomic Force Microscopy

The films were analyzed visually according to Nogueira et al. [31] with regard to homogeneity (presence of insoluble particles), film-forming capacity (continuity and apparent cracks), and ease of handling.

The surface and cross-section of the films were performed using a scanning electron microscope (Tabletop Microscope Hitachi, TM 300, Tokyo, Japan) operating at an acceleration voltage of 15 kV. To evaluate the surface and cross-section, the films were freeze dried and fixed to the equipment support with double-sided conductive copper tape (Ted Pella, Inc.—Redding, CA, USA).

Atomic force microscopy (AFM) was performed in an atomic force microscope (Solver Next SPM, NT-MDT, Zelenograd, Russia) in intermittent mode with a contact force of 5 N/m, scanning speed of 0.5 Hz using a tip (TAP 150AL-G, Budget Sensors Company, Sofia, Bulgaria) with resonance frequency of 150 kHz. The image area was 50 µm^2^ and the average roughness was determined using Image Analysis 3.2.5 software. 

#### 2.5.2. UV/Visible Barrier, Transparency, and Luminosity

The ultraviolet/visible (UV/Vis) barrier was determined according to Fang et al. [32] using a spectrophotometer (Perkin Elmer, LAMBDA 35, Boston, MA, USA) with a wavelength ranging from 200 to 800 nm. Transparency was calculated according to Equation (1), reported by Han and Floros [33]. The luminosity (L*) was determined using a colorimeter (HunterLab, Miniscan XE plus, Reston, VA, USA):

(1) $$TR=\frac{Abs_{600}}{x}$$

where: TR is the film absorbance at 600 nm; x (mm) is the film thickness. 

#### 2.5.3. Thickness and Mechanical Properties

Thickness was determined using a digital micrometer (Mitutoyo, Absolute, Interface IT-016U, Kawasaki, Japan) performing 10 random measurements in the films. The tensile strength (TS) and elongation (E) were determined using a texturometer (Stable Microsysems SMD, TA XT2, Godalming, UK) according to ASTM D882-10 [34]. Film samples (100 mm × 25 mm) were positioned between the claws (100 mm apart) with a test speed of 10 mm s^−1^, and the films were pulled until rupture. The TS and E were obtained from the tensile curve (MPa) by elongation (%).

#### 2.5.4. Moisture, Solubility in Water, and Contact Angle 

Moisture was determined according to Gontard et al. [35], where film samples (2 cm × 2 cm) were oven dried (Fanem, 515, Sao Paulo, Brazil) at 105 °C for 24 h and the moisture content was determined considering the samples mass loss.

To determine solubility, samples (2 × 2 cm) were dried at 105 °C (24 h) and transferred to beakers with 50 mL of distilled water, submitted to shaking (100 rpm) in a Shaker incubator (Marconi, MA 420, Piracicaba, Brazil) for 24 h [35]. Subsequently, the samples were again oven dried (Fanem, 515, Sao Paulo, Brazil) at 105 °C for 24 h and the soluble material was determined according to Equation (2):

(2) $$Solubility\left(\%\right)=\left(\frac{m_{i}-m_{f}}{m_{i}}\right)$$

where: $m_{i}$ is the (g) sample initial dry mass and $m_{f}$ is the (g) sample final dry mass.

The contact angle was determined using a tensiometer (Attension Theta Lite, KSV Instruments, Filderstadt, Germany) according to Abdollahi et al. [36], which consisted of fixing the film sample (2 × 3 cm) to the base of the equipment and depositing a drop of ultrapure water (5 µL) onto the film surface. Images were recorded after 10 s and digital images were analyzed using the Attension Theta Lite software (Version 4.1.9.8).

#### 2.5.5. Fourier Transform Infrared (FTIR) Spectroscopy

Fourier transform infrared (FTIR) spectroscopy was performed using a spectrometer (Spectrum One, Perkin Elmer, Boston, MA, USA), and the data were analyzed by FTIR Spectrum Software. A total of 16 scans were carried out in the spectral range from 650 to 4000 cm^−1^ with a 2 cm^−1^ resolution.

#### 2.5.6. Antimicrobial Activity

The zone of inhibition test was performed according to Section 2.2.2 in order to verify the films antimicrobial activity. For this, film discs (5.5 mm in diameter) were placed on a plate containing the sown inoculum. This test was performed using only the bacteria that were inhibited in the zone of inhibition test for copaiba oil (Section 2.2.2). After the incubation period, microbial growth, and the formation of ≥8 mm inhibition zones were observed [28,29].

### 2.6. Statistical Analysis

The InfoStat^®^ software (version 2018d) was used to calculate the analysis of variance (ANOVA). The Tukey test was performed to determine the difference between the means at a 95% confidence level.

## 3. Results and Discussion

### 3.1. Caracterization of Copaiba Oil

#### 3.1.1. Chemical Composition of Copaiba Oil

Table 1 shows the chemical profile of copaiba oil, in which it was possible to identify 30 sesquiterpene compounds, with β-caryophyllene being the major compound, representing 48.29% of the total composition. According to studies reported in the literature, β-caryophyllene is considered the main compound of copaiba oil [37,38].

Other predominant compounds in copaiba oil were the sesquiterpenes α-guaiene (11.58%), β-bisabolene (5.27%), and α-copaene (5.01%), which corroborate with studies of different species of *Copaifera* sp. [38,39]. According to Leandro et al. [15], sesquiterpenes are the main components found in copaiba oil, which may represent more than 90% of its composition. The chemical composition of this oil can vary between species of the *Copaifera* genus and this variation is generally associated with the climate, soil, and harvest area [40].

Barbosa et al. [41] identified 35 different constituents in copaiba oil extracted from *Copaifera multijuga*, with a high predominance of sesquiterpenes (88.55 to 98.05%). Herrero-Jáuregui et al. [42] reported that, among the identified sesquiterpenes, the predominant ones in the *Copaifera reticulada* D. oil were β-caryophyllene, trans-α-bergamotene, and β-bisabolene. Emerenciano et al. [43] reported that the copaiba oil obtained from *Copaifera multijuga* H. presented 73.1% sesquiterpenes, 16.9% diterpenes, and 10.5% fatty acids in its composition.

#### 3.1.2. Zone of Inhibition Test

**Figure 1:**
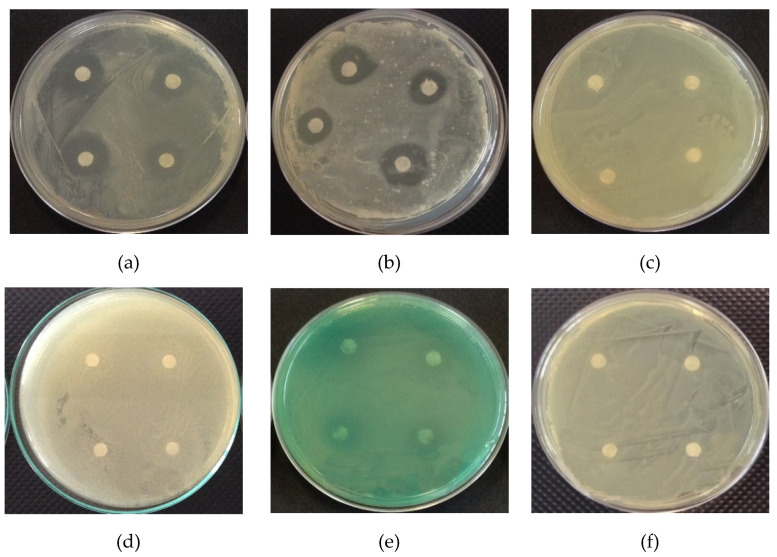
Antimicrobial activity of copaiba oil against: (**a**) *Bacillus subtilis*, (**b**) *Staphylococcus aureus*, (**c**) *Enterobacter aerogenes*, (**d**) *Escherichia coli*, (**e**) *Pseudomonas aeruginosa*, and (**f**) *Salmonella typhimurium*.

Figure 1 shows the antimicrobial activity of copaiba oil against gram-positive bacteria (*Bacillus subtilis* and *Staphylococcus aureus*) and gram-negative bacteria (*Enterobacter aerogenes*, *Escherichia coli*, *Pseudomonas aeruginosa*, and *Salmonella typhimurium*).

In Figure 1a,b, the formation of an inhibition zone against the studied gram-positive bacteria was observed, with zones of 17.25 ± 0.19 and 16.58 ± 0.19 mm against *B. subtilis* and 16.58 ± 0.19 mm against *S. aureus*, respectively, demonstrating a potential for application in biodegradable films.

The cell wall of gram-positive bacteria is made up of teichoic and lipoteichoic acids, which can assist in the penetration of hydrophobic components of essential oils into cells [44]. Gram-negative bacteria have a lipopolysaccharide outer membrane (LPS), which replaces most phospholipids in the outer membrane and limits the diffusion rate of hydrophobic compounds in the polysaccharide layer [45]. For this reason, gram-negative bacteria are more resistant to the effects of essential oils than gram-positive bacteria [46], which is possibly related to the non-formation of inhibition zones against the gram-negative bacteria studied (Figure 1c–f).

Pacheco et al. [47] evaluated the antimicrobial activity of different varieties of copaiba (*Copaifera* sp.) oils and reported that the levels of inhibition against *S. aureus* and *B. subtilis* bacteria varied. This difference was attributed to the chemical composition of oils, and none of the oils evaluated showed antimicrobial activity against gram-negative bacteria. Morelli et al. [18] reported that copaiba oil had an antibacterial effect against *B. subtilis*, forming an inhibition zone of 11 mm.

According to Leandro et al. [15] and Morelli et al. [18], copaiba oil bioactivity is confirmed mainly by the presence of sesquiterpenes, with an emphasis on β-caryophyllene however, the antimicrobial effects are obtained from the synergy between the compounds. Goren et al. [48] attributed β-caryophyllene to the antimicrobial activity against *E. coli* and *S. aureus*, forming zones of 20 and 24 mm. According to Dahham et al. [49], β-caryophyllene presented an antimicrobial effect against *B. cereus*, *B. subtilis*, *S. aureus*, and *E. coli*.

#### 3.1.3. Minimum Inhibitory Concentration (MIC)

Table 2 shows the inhibition zone for the minimum inhibitory concentration (MIC) of copaiba oil against *Bacillus subtilis* and *Staphylococcus aureus* bacteria.

The copaiba oil showed an inhibition zone of 7.75 to 15.00 mm for *B. subtilis* and of 6.86 to 15.00 mm for *S. aureus*, the increase in the oil concentration led to an increase in the inhibition zone. According to Ponce et al. [28] and Palmeira et al. [29], a satisfactory inhibitory activity is observed in zones with a ≥ 8 mm diameter therefore, 12% of copaiba oil was selected as MIC.

Mendonça and Onofre [27] evaluated the antimicrobial activity of copaiba oil (*Copaifera multijuga*) and its 12.5% MIC was efficient against *S. aureus* (9 mm inhibition zone). Santos et al. [50] reported that different copaiba oils (*C. martii*, C. *officinalis*, and C. *reticulata*) showed antimicrobial activity against gram-positive bacteria.

### 3.2. Caracterization of Films

#### 3.2.1. Visual Analysis, Scanning Electron Microscopy, and Atomic Force Microscopy

**Figure 2:**
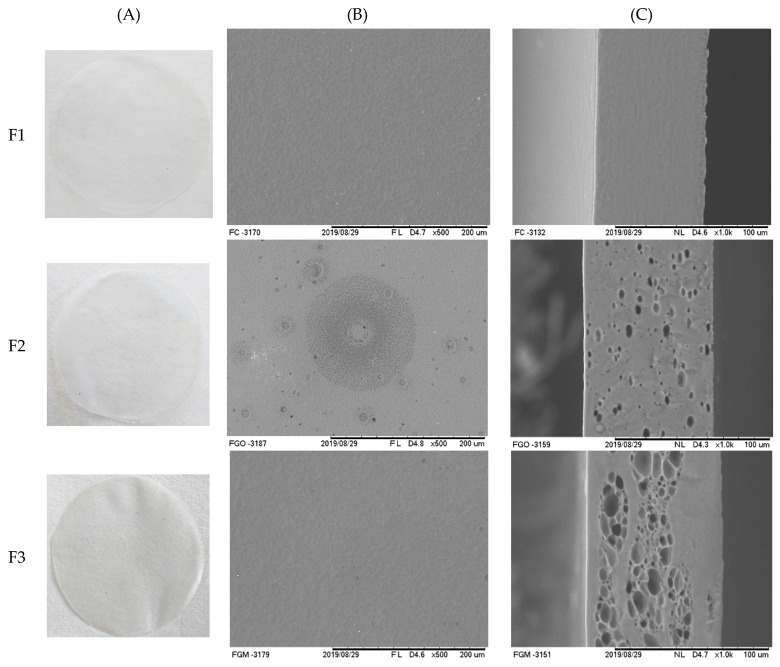
Mangarito starch-based films: (**A**) Visual aspect, scanning electron microscopy (SEM) of (**B**) surface (images with 500x magnification) and (**C**) cross-section (images with 1000x magnification), (**F1**) control film, (**F2**) film with copaiba oil by direct incorporation, and (**F3**) film with microencapsulated copaiba oil.

Figure 2A shows the films based on mangarito starch with plasticizer, incorporated with copaiba oil directly and in a microencapsulated form. In general, the films presented a homogeneous surface and no insoluble particles. According to Mali et al. [51], when glycerol is incorporated into starch, molecular interactions occur due to hydrophilic affinity and the polymeric matrix undergoes structural modifications, which makes it less dense, facilitating the movements of the polysaccharide chains.

The microstructures of mangarito starch-based films were evaluated according to the incorporation of copaiba oil, directly and microencapsulated (Figure 2B,C), in which the control film (Figure 2F1(B,C)) presented a homogeneous and smooth structure, without pores or fissures, which corroborates with what was observed visually, indicating that the starch granules were completely broken and the amylose/amylopectin molecules gelatinized [52]. One of the properties of starch is its good ability to form cohesive films when produced under specific conditions [1]. In the film incorporated with copaiba oil directly (Figure 2F2(B,C)), a discontinuous matrix was formed, which possibly promoted the formation of heterogeneous zones. The oil may have made it difficult to aggregate the polymeric chain during the drying process, forming concave and convex irregularities [53].

Brandelero et al. [17] reported that the incorporation of copaiba and lemongrass oils changed the structure of films based on cassava starch, polyvinyl alcohol, and sodium alginate and through the micrographs, they detected the presence of pores and cracks in the film matrix. During the drying process, the essential oils dispersed into the film-forming solution may suffer phenomena that destabilize the emulsion, such as flocculation and coalescence, in which the oil droplets partially move to the surface, providing volatilization and, consequently, the appearance of holes [54]. The microcapsules serve as protection and prevent these phenomena from happening to the encapsulated oil.

In the films incorporated with microencapsulated copaiba oil (Figure 2F3(B)), the formation of microcapsule clusters was observed, mainly distributed in the lower part of the film, indicating that they may have moved to the bottom of the plate during the drying process. Possibly, the microparticles remained intact after the production and drying process of the films and preserved the cohesive regions formed by the polymer (Figure 2F3(B,C)).

Martínez-Ortiz et al. [55] reported that ascorbic acid microcapsules remained dispersed in a small area of the film, being better observed when the microcapsule concentration was higher. The authors also suggested that the increase in the number of microparticles contributed to the increase in the films’ mechanical properties. Moraes Crizel et al. [56] reported that a higher concentration of papaya peel microparticles smoothed the surface of gelatin films, making them similar to the control film. 

**Figure 3:**
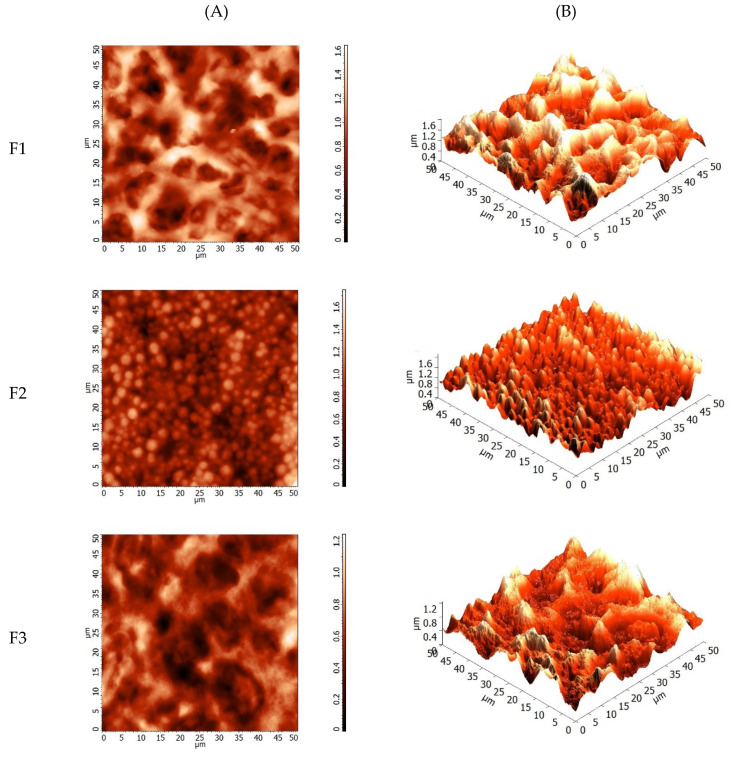
Atomic force microscopy images of the surface in the 2 (**A**) and 3 (**B**) dimensions of mangarito starch-based films: (**F1**) Control film, (**F2**) film with copaiba oil by direct incorporation, and (**F3**) film with microencapsulated copaiba oil.

Figure 3 shows the atomic force microscopy of the films of mangarito starch incorporated with copaiba oil directly and microencapsulated.

Figure 3F1(A,B) shows that the control film had a more irregular surface than the others, with an average roughness of 232.23 ± 15.87^a^ nm, differing significantly. The film incorporated with copaiba oil directly Figure 3F2(A,B) showed a lower roughness (175.41 ± 22.21^b^ nm) due to the distribution of oil over the entire film surface. On the other hand, the incorporation of microencapsulated copaiba oil (Figure 3F3(A,B)) promoted more accentuated regions in comparison to the control film, but showed more evident irregularities in relation to the film incorporated with oil directly and presented an average roughness of 181.84 ± 25.29^b^ nm however, it did not differ significantly from the film incorporated with copaiba oil directly.

The reduction in the roughness of films incorporated with oil directly may be associated with the oil liquid state, which filled in the irregularities of the polymer matrix during the drying process, resulting in more regular surfaces [57]. As for the incorporation of microparticles, the reduction in roughness in relation to the control film may be associated with the presence of gelatin and arabic gum as a coating for the microparticles, which promoted a reduction in the polymer/microparticle interface.

As observed in Figure 3(F2,F3) the addition of oil directly or microencapsulated favored the change in the surface of the films. The films containing copaiba oil in direct or microencapsulated form was less rough when compared to the control, which can influence the antimicrobial activity of the film. According to Ghasemlou et al. [57] the fact that the essential oil of *Zataria multiflora* B. is more distributed throughout the polymeric matrix of corn starch films, it may be responsible for its greater antimicrobial activity.

#### 3.2.2. UV/Visible Barrier, Transparence, and Luminosity 

**Figure 4:**
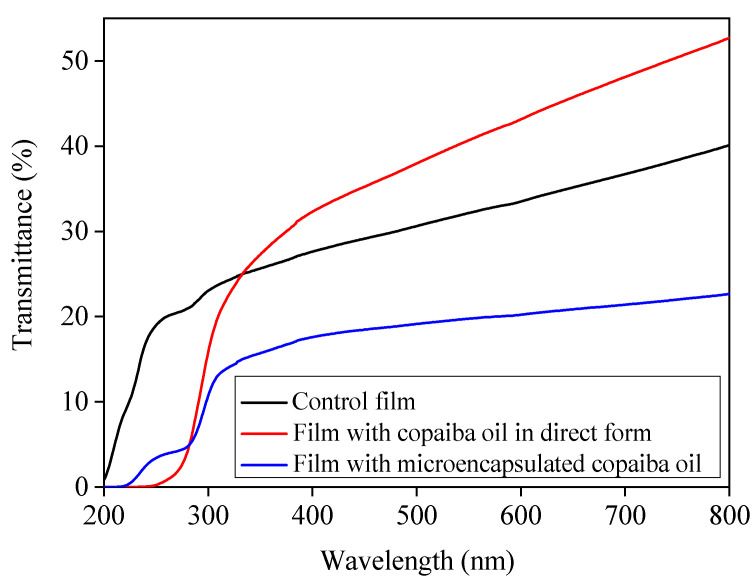
Effect of the incorporation of copaiba oil, directly and microencapsulated, on the wavelength from 200 to 800 nm due to the transmittance of mangarito starch-based films.

In Figure 4, it was observed that the film added with microencapsulated copaiba oil showed lower transmittance in comparison to the others, indicating that the form in which the copaiba oil was incorporated influenced the UV/Vis barrier. Similarly, Al-Hassan and Norziah [58] reported that sago starch/fish gelatin films showed higher energy absorption than films without gelatin. According to Šuput et al. [59], the light absorption in starch-based films is proportional to the addition of essential oil in the visible range (350 to 800 nm). When the oil is added at an appropriate concentration, the films can be applied as food packaging capable of controlling oxidative changes.

The film incorporated directly with copaiba oil also showed lower transmittance up to ~330 nm in relation to the control film however, the transmittance was higher in the visible region, possibly due to the form of incorporation applied, which can cause the formation of less homogeneous films. The transmittance values obtained for the three formulations were lower than those reported for the oriented propylene and the low-density polyethylene, which are plastics used as packaging [60]. Sartori and Menegalli [61] reported that films of green banana starch showed less light transmittance after the incorporation of lipid microparticles of ascorbic acid. The transmittance of the films incorporated with microencapsulated oil were also lower than that of the gelatin-based films incorporated with microparticles of chitosan [62].

From the absorbance of samples at 600 nm as well as the thickness, it was possible to determine film transparency (Table 3). The film incorporated with microencapsulated copaiba oil showed greater transparency (8.89%), followed by the control film (6.18%), and the one incorporated with copaiba oil directly (4.88%). The greater transparency of the film incorporated with microencapsulated copaiba oil can be possibly attributed to the gelatin used for the coating of the microparticles. According to Fakhouri et al. [63], gelatin-based films are more transparent while films added with lipids are more opaque.

Tumwesigye et al. [64] produced peeled and intact bitter cassava films and reported that the films showed a transparency of 3.64% and 11.94%, respectively. The transparency of a film is an impact factor in the appearance of a packaged product, apart from helping consumers to identify the product before purchasing it [22].

According to Table 3, the films showed luminosity (L*) ranging from 90.84 to 90.13, and the incorporation of oil and microparticles reduced this parameter. The film incorporated directly with copaiba oil did not differ significantly from the others, indicating that copaiba oil did not interfere with the luminosity. On the other hand, the film incorporated with microencapsulated copaiba oil differed (p < 0.05) from the control film. Šuput et al. [59] also observed a reduction in the luminosity of corn starch-based films after the addition of essential cumin oil (L* = 95.32 to 93.25) and oregano (L* = 95.32 to 91.36).

#### 3.2.3. Thickness and Mechanical Properties 

Table 3 shows the thickness and mechanical properties (tensile strength and elongation) of mangarito starch-based films incorporated with copaiba oil directly and in microencapsulated form. The films based on mangarito starch (control film) showed no difference (p > 0.05) in thickness (~0.07 mm) in comparison to the films produced with the oil incorporated directly and in microencapsulated form, indicating that the mass control was efficient during the production process.

Overall, there was a reduction in the tensile strength and elongation of the films based on mangarito starch after the incorporation of copaiba oil in comparison to the control film and the one added with microparticles. This reduction is possibly related to the heterogeneity observed in the polymeric matrix after incorporating the oil. According to Bonilla et al. [65] the presence of oil can weaken the intermolecular forces of the polymer chain and make the film matrix less resistant. Brandelero et al. [17] also reported that the incorporation of copaiba oil reduced the tensile strength and elongation of films made of cassava starch, polyvinyl alcohol, and sodium alginate, and attributed this to the structural modification of the films due to the presence of the oil, which resulted in a discontinuous matrix.

**Figure 5:**
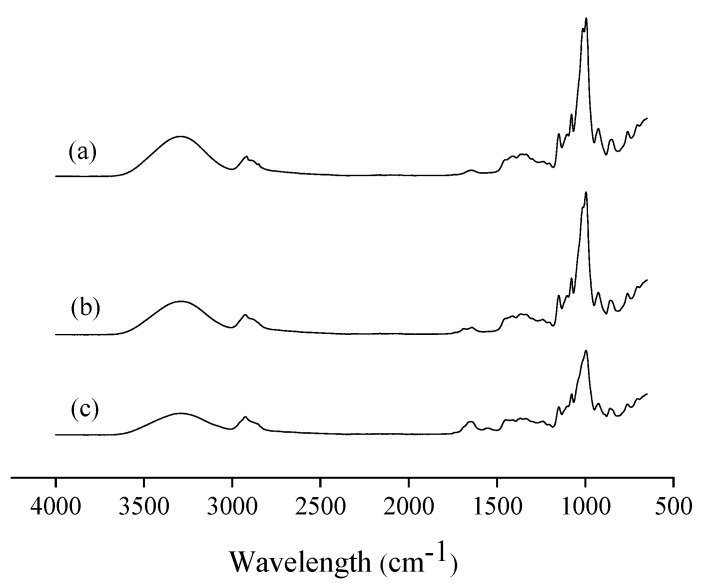
The effect of incorporating microencapsulated copaiba oil directly on the infrared spectra of mangarito starch-based films: (**a**) Control film, (**b**) film incorporated with copaiba oil directly, and (**c**) film incorporated with microencapsulated copaiba oil.

Song et al. [19] attributed the reduced tensile strength of films based on corn and wheat starch, and the formation of a heterogeneous and discontinuous structure, to the incorporation of lemon essential oil into the polymeric matrix. The same was reported by Pelissari et al. [66] for films based on cassava starch, glycerol, and chitosan after the incorporation of oregano oil. The film incorporated with microparticles of copaiba oil showed no difference (p> 0.05) in tensile strength from the control film, indicating good interaction between the microparticles and starch. An increase (p <0.05) in elongation was also observed (Table 4), indicating that the incorporation of microparticles caused a plasticizing effect in the film due to the formation of new bonds, as can be seen in Figure 5c.

Wang et al. [67] found that the incorporation of orange peel oil nanoparticles into corn starch-based films led to the formation of new hydrogen bonds with the hydroxyl group, altering the properties of the film and increasing the tensile strength and elongation, in comparison to control films (starch only).

#### 3.2.4. Moisture, Solubility, and Contact Angle

According to Table 3, there was a decrease in the moisture of the films with microparticles in comparison to the control film and those with the addition of oil. Song et al. [19] observed a similar behavior for corn starch-based films incorporated with lemon essential oil. Dashipour et al. [68] reported that the moisture content of the carboxymethylcellulose-based film decreased significantly when *Zataria multiflora* essential oil was incorporated and attributed this to the reduction of the films hydrophilic nature.

The addition of copaiba oil, directly and microencapsulated, reduced (p < 0.05) the solubility of the films (Table 3). This can be attributed to the reduction of the films hydrophilic character caused by the interaction between the oil components and hydroxyl groups of the polymeric matrix, which become less available and consequently improves the films resistance to water [69]. This corroborates with the results obtained in the contact angle (Table 3), in which the films with copaiba oil and microparticles presented hydrophobic characteristics. Furthermore, the addition of microparticles reinforces the film structure, reducing the spaces in the polymer/microparticle interface, and promotes the diffusion of solute in the film [56].

One of the biggest challenges of applying polysaccharides to films for food packaging is high sensitivity to water [70]. The value of the contact angle allows monitoring the hydrophilic and hydrophobic character of the film surface therefore, the contact angle increases according to the hydrophobicity of the surface [71]. Hydrophilic surfaces have contact angle values of <20°, while hydrophobic surfaces have values of >70° [52].

According to Table 3, the degree of hydrophilicity of mangarito starch-based films was reduced with the incorporation of copaiba oil, directly and microencapsulated, which differed (p < 0.05) between the formulations. Films with copaiba oil incorporated directly showed a greater contact angle (92.45°), followed by films with microencapsulated copaiba oil (81.78°), and the control film (47.19°). This can be explained by the incorporation of hydrophobic substances present in the oil [71].

Slavutsky and Bertuzzi [52] reported that the terpenes present in the oil can interact with starch through weak bonds, such as hydrogen bond, guaranteeing adhesion between these materials. Carboxymethyl cellulose-agar biocomposite film with *Satureja hortensise* essental oil [36] and sodium alginate films with carvacrol microparticles [72] presented a greater contact angle, which is attributed to the hydrophobic nature of the added substances.

#### 3.2.5. Fourier Transform Infrared (FTIR) Spectroscopy

The infrared spectra of mangarito starch-based films incorporated with copaiba oil directly and microencapsulated are shown in Figure 5, where characteristic starch peaks are observed [73,74].

In the 4000 to 2500 cm^−1^ region, the spectra show absorption bands at 3296 cm^−1^, which correspond to the −OH stretch formed with hydroxyl groups of starch and glycerol [66,73] and between 2918 and 2926 cm^−1^, attributed to the absorption of alkanes due to the stretching of C–H [75]. While in the region from 2000 to 1500 cm^−1^, absorption bands were observed between 1642 and 1648 cm^−1^, which corresponds to the angular deformation of −OH bonds [74]. A small peak at 1553 cm^−1^ was observed only in the film with microencapsulated oil (Figure 5c), characteristic of the amino group [66]. This peak can be related to the microparticle covering material, since Dammak et al. [62] reported that gelatin films showed an amino band at 1537 cm^−1^.

The 1500 cm^−1^ region corresponds to the digital fingerprint of the spectrum, in which there is a large number of absorptions due to a variety of vibrations of simple bonds [75]. The absorption bands at 1078, 1077, 950, 996, and 926 cm^−1^ correspond to C–OH and CH_2_ deformations [74]. According to Lima et al. [74], the absorption bands in the region of 1200 to 1000 cm^−1^ are characteristic of starch, attributed to vibrations of axial deformation of C–O and O–C–O.

Pelissari et al. [66] reported absorption regions at 3304, 2922, 1164, 1077, 1019, and 921 cm^−1^ for cassava starch and glycerol films, which are very similar to the bands obtained in the present study. In general, no difference in peaks or displacement of absorption bands was identified. Brandelero et al. [17] reported that copaiba and lemongrass essential oils did not promote changes in the intensity and position of peaks and bands, indicating that there were no chemical interactions between the essential oils and film matrix and, therefore, the bioactive effects of essential oils were not compromised after mixing.

#### 3.2.6. Antimicrobial Activity

Table 4 shows the inhibition zones corresponding to the antimicrobial activity of films containing copaiba oil added directly or in microencapsulated form against *Bacillus subtilis* and *Staphylococcus aureus*.

The control film did not show antimicrobial activity against the studied bacteria, since the mangarito starch does not have antimicrobial activity. However, the incorporation of copaiba oil into the tested concentrations attributed antimicrobial properties to the films.

The film incorporated with copaiba oil directly allowed the formation of inhibition zones of 9.75 mm, close to the value obtained with 12% pure copaiba oil in the MIC analysis (9.88 mm) for *B. subtilis*, indicating that the oil bioactivity was maintained even after being mixed with the filmogenic solution and submitted to the drying process. However, the bioactive compounds of the oil that remained in the film were not sufficient to inhibit *S. aureus*, as this was more resistant to inhibition.

The film with microencapsulated copaiba oil was able to inhibit the two bacteria tested, in which the antimicrobial effect of copaiba oil was enhanced due to being microencapsulated, since the microparticle film showed an inhibition zone of 11.38 mm against *B. subtilis* and 8.8 mm against *S. aureus*, which was higher than the MIC test. Possibly, during the film production process, the microparticle protected the compounds with antimicrobial activity from the copaiba oil.

According to Medeiros et al. [21], the efficiency of the films in acting as active packaging is influenced not only by the concentration of oil, but also by the diffusion of the bioactive compounds into the medium, which in return depend on the interactions between the components, as well as their disposition inside the film. Microencapsulation of bioactive compounds is a technology that allows isolating and preserving compounds from inactivation by external agents [30]. Thus, the microcapsule has the function of stabilizing and protecting the compounds against deterioration, losses due to volatilization, or unwanted interaction with other components [76,77]. Microparticles produced by complex coacervation have excellent controlled release characteristics and are more resistant to heat [78]. Morelli et al. [18] reported an inhibition zone of 22 mm against *B. subtilis* using paper and poly (lactic acid) film incorporated and coated, respectively, with 20% copaiba oil.

## 4. Conclusions

Copaiba oil has antimicrobial activity, with β-caryophyllene (48.29%) as the major compound, and a minimum inhibitory concentration of 12%, against *B. subtilis and S. aureus*. It was possible to produce *Xanthosoma mafaffa* Schott starch-based films with the oil incorporated directly as well as microencapsulated. The incorporation of this oil in microencapsulated form, in comparison to the direct incorporation, increased the tensile strength, elongation, and moisture, and reduced the transmittance. In relation to the control film, both forms of oil incorporation reduced the solubility and increased the contact angle of the films. Thus, mangarito starch-based films added with copaiba oil could be considered a packaging alternative for food application.

## Figures and Tables

**Table 1:** Chemical profile of copaiba oil by gas chromatograph coupled with mass spectrometry (GC-MS) at retention time (RT), calculated index (CI), and literature index (LI).

RT (min)	CI	LI	Compound	Content (%)
23.370	1333	1335	δ-Elemene	0.42
24.121	1350	1350	α-Longipinene	0.73
25.034	1372	1374	α-Copaene	5.01
25.697	1389	1389	β-Cubenene	1.79
25.975	1394	1398	Cyperene	0.36
26.979	1408	1408	β-Caryophyllene	48.29
27.210	1424	1430	β-Copaene	0.11
27.428	1430	1432	trans-α-Bergamotene	0.96
27.596	1437	1437	α-Guaiene	11.58
27.820	1439	1440	cis-β-Farnesene	0.53
28.001	1444	1445	epi-β-Santalene	0.21
28.248	1450	1452	α-Humulene	0.77
28.500	1456	1458	allo-Aromadendrene	4.56
28.845	1464	1464	9-epi-Caryophyllene	0.02
29.042	1469	1469	β-Acoradiene	0.23
29.333	1476	1478	γ-Muurolene	2.62
29.502	1481	1483	α-Amorphene	4.83
29.745	1487	1484	Germacrene D	1.10
30.110	1496	1496	Viridiflorene	0.40
30.235	1499	1502	β-Guaiene	1.08
30.339	1506	1505	β-Bisabolene	5.27
30.9 61	1510	1513	γ-Cadinene	0.64
31.057	1520	1522	δ-Cadinene	3.50
31.570	1533	1537	α-Cadinene	0.10
31.617	1532	1532	γ-Cuprenene	2.60
32.295	1552	1559	Germacrene B	0.76
35.314	1640	1640	epi-α-Muurolol	0.30
35.648	1645	1645	Cubenol	0.63
35.932	1649	1649	β-Eudesmol	0.39
36.191	1652	952	α-Cadinol	0.31

**Table 2:** Effect of different concentrations of copaiba oil on the formation of inhibition zones (mm) against the growth of *B. subtilis* and *S. aureus* bacteria.

Concentration (%)	*B. subtilis*	*S. aureus*
3	7.75 ± 0.07 ^d^	6.86 ± 0.06 ^c^
6	8.86 ± 0.07 ^cd^	7.00 ± 0.09 ^c^
12	9.88 ± 0.12 ^c^	8.43 ± 0.28 ^bc^
25	9.88 ± 0.04 ^c^	9.50 ± 0.14 ^bc^
50	12.75 ± 0.09 ^b^	11.13 ± 0.27 ^b^
100	15.00 ± 0.23 ^a^	15.00 ± 0.18 ^a^
Positive control	27.00 ± 0.00	20.00 ± 0.00
Negative control	Nd	Nd

^a–d^ Different lower case letters in the same column indicate significant difference (*p* < 0.05). Nd (not detected).

**Table 3:** Effect of the incorporation of copaiba oil, directly and microencapsulated, on the optical, mechanical and barrier properties of mangarito starch-based films.

Analyzes	F1	F2	F3
Luminosity (L*)	90.84 ± 0.56 ª	90.49 ± 0.56 ^ab^	90.13 ± 0.24 ^b^
Transparency (%)	6.18 ± 0.45 ^b^	4.88 ± 0.30 ^c^	8.89 ± 0.23 ^a^
Thickness (mm)	0.074 ± 0.002 ^a^	0.079 ± 0.001 ^a^	0.077 ± 0.002 ^a^
Stress at break (MPa)	3.46 ± 0.12 ^a^	2.41 ± 0.33 ^b^	3.42 ± 0.32 ^a^
Elongation (%)	16.44 ± 1.00 ^b^	15.47 ± 2.53 ^b^	20.05 ± 2.16 ^a^
Moisture (%)	21.57 ± 2.93 ^a^	19.65 ± 0.99 ^a^	13.93 ± 2.79 ^b^
Solubility in water (%)	20.00 ± 0.65 ^a^	15.89 ± 1.07 ^b^	16.87 ± 0.68 ^b^
Contact angle (°)	47.19 ± 4.89 ^c^	92.45 ± 6.67 ^a^	81.78 ± 2.79 ^b^

F1: Control film, F2: Film with copaiba oil incorporated directly, and F3: Film with microencapsulated copaiba oil. ª a statistical analysis. ^a–c^ Different lowercase letters in the same row indicate significant difference (*p* < 0.05).

**Table 4:** The effect of the incorporation of copaiba oil, directly and microencapsulated, on the formation of inhibition zones (mm) against the growth of *Bacillus subtilis* and *Staphylococcus aureus* bacteria.

Formulation	*B. subtilis*	*S. aureus*
F1	Nd	Nd
F2	9.75 ± 0.07	Nd
F3	11.38 ± 0.11	8.80 ± 0.04
12% of copaiba oil *	9.88 ± 0.12	8.43 ± 0.28

F1: Control film, F2: Film with copaiba oil added directly, and F3: Film with microencapsulated copaiba oil. Nd (not detected). * Data obtained in the minimum inhibitory concentration (MIC) test.
